# Leaving no one behind: Disability and HIV prevention, knowledge among adults in a population cohort in Uganda

**DOI:** 10.4102/ajod.v13i0.1497

**Published:** 2024-12-05

**Authors:** Joseph O. Mugisha, Ronald Makanga, Beatrice W. Kimono, Ivan Kasamba

**Affiliations:** 1 Medical Research Council/Uganda Virus Research Institute and London School of Hygiene and Tropical Medicine, Uganda Research Unit, Entebbe, Uganda

**Keywords:** HIV, disability, adults, general population, Uganda

## Abstract

**Background:**

People with disability are a vulnerable population and are at a high risk of acquiring human immunodeficiency virus (HIV) infection.

**Objectives:**

We investigated the association between severity of disability and not having knowledge of any HIV prevention method among adults in Uganda.

**Method:**

Between January 2015 and December 2015, data were collected within a general population in Uganda, on six domains of disability based on the Washington Group Short Set on Functioning. In addition, routine data on socio-demographic factors and other HIV-related data were collected among adults aged 16 years and above. A continuum of functioning was developed: without disability, mild, moderate and severe. Bivariate and multivariate associations of disability and not knowing any HIV prevention method were fitted using logistic regression models.

**Results:**

A total of 3331 adults (60.4% female) were included. Of these, 14.5% (*n* = 482) were classified as having either moderate or severe disability, and this proportion exponentially increased with age (*p* < 0.001). Overall, 4.2% (*n* = 140) reported not knowing any HIV prevention method, with a slightly higher proportion among females than males (3.1% in males, 4.9% in females). Compared to people with no disability, those with moderate or severe disability were 5 times more unlikely to know any HIV prevention methods (adjusted odds ratio = 5.45, 95% confidence interval = 3.25–9.13, *p* < 0.001). Additionally, the combined effect of moderate and severe disability and none/incomplete primary education increased the likelihood of not knowing HIV prevention methods over and beyond their separate effects (*p* < 0.001).

**Conclusion:**

Effective HIV prevention strategies must integrate best practices that target people with disabilities.

**Contribution:**

These findings contribute to the evidence of the lack of HIV prevention knowledge among people with disabilities in general populations.

## Introduction

Despite declining trends in human immunodeficiency virus (HIV) infections globally, ongoing transmission continues with substantial risk for HIV among some vulnerable sub-populations. The recent Joint United Nations Programme on HIV/AIDS (UNAIDS) report (UNAIDS 2023) (Van Schalkwyk et al. 2024) on the global HIV statistics indicated that the steepest drops in numbers of new HIV infections was among children (0–14 years). The report also indicates that declines in the numbers of new HIV infections were strongest in sub-Saharan Africa (SSA). However, there are key sub-populations (Kiwanuka et al. 2014; Shisana et al. 2015) including adolescent girls and young women who still have extremely high risk of HIV infections in SSA (Jin, Restar & Beyrer 2021; Kasamba et al. 2023; Karim & Baxter 2019). People with disabilities might experience similar HIV risks because of the broad range of barriers in accessing HIV prevention information and strategies including social and structural barriers (Hashemi et al. 2022; Schenk et al. 2020; Tun et al. 2016).

In Uganda, HIV incidence started falling in the 1990s and has continued to fall, but HIV incidence remains high in females compared to males (Grabowski et al. 2017b), and a big proportion of new HIV infections remains among younger people aged 15–30 years (Kasamba et al. 2023). In addition, there are still some key populations including the fisher folk and female sex workers where the HIV incidence remains disproportionally high compared to other populations (Dellar, Dlamini & Karim 2015; Jin et al. 2021; Papworth et al. 2013). Despite the barriers to accessing HIV prevention and care services among people with disabilities, population level data on risk, HIV knowledge and access to HIV preventive and treatment services remain scarce.

The World Health Organization (WHO) estimates that approximately 1.3 billion people, representing about 16% of the global population experience a significant disability. A bigger number (approximately 2.4 billion) have difficulties with functioning and would benefit from rehabilitation (Cieza et al. 2020). National estimates suggest that 11% – 13% of the population in Uganda experience at least some functional difficulty with vision, hearing, mobility, communication, cognition and/or self-care (Eide, Nanono & Omona 2020). The number of people with disabilities is likely to be growing because of an increase in non-communicable diseases (NCDs) and increasing number of older people. Persons with disabilities are likely to die earlier compared to people without disabilities, have poorer health and experience more limitations in everyday functioning than others (Ahumuza et al. 2014; Eide, Nanono & Omona 2021; Mulumba et al. 2014).

By 2017, data from SSA suggested an increased risk of HIV infection of 1.48 times in men with disabilities and 2.21 times in women with disabilities compared to men and women without disabilities (De Beaudrap, Mac-Seing & Pasquier 2014; De Beaudrap et al. 2016). Despite an increased risk of HIV, people with disabilities have been neglected in all sectors responding to HIV, including HIV prevention. There are several factors that increase the risk of HIV in persons with disabilities. These include stigma and discrimination (Nixon et al. 2014; Tun & Leclerc-Madlala 2017; UNICEF 2012), exclusion of persons with disabilities from violence prevention, inaccessibility of health care facilities and other HIV prevention facilities (Hanass-Hancock & Alli 2015), exclusion from sexual education (International 2013) and increased economic vulnerability (Banks, Kuper & Polack 2017).

Within the Ugandan context, data on HIV and disability within general populations are scarce. One study based on the 2011 Uganda health and demographic survey (Abimanyi-Ochom et al. 2017) established comparable levels of knowledge on HIV and acquired immunodeficiency syndrome (AIDS) for those with and those without disabilities in relation to HIV transmission during delivery (93.9% and 93.3%, respectively) and through breastfeeding (89.9% and 90.6%, respectively). However, people with disabilities had misconceptions of risk of HIV infection and how HIV is transmitted compared to those without disabilities. This study did not look at many other variables related to HIV risk and other HIV prevention methods among people with disabilities.

Currently, the methods undertaken for HIV prevention in Uganda include prevention of mother to child transmission, safe male surgical circumcision, treatment for prevention, pre- and post-exposure prophylaxis, in addition to the abstinence, be faithful and condom use (ABC) strategies. In 2021, UNAIDS set the 95–95–95 targets, that include HIV testing, treatment and viral suppression that aimed to close gaps in HIV treatment coverage and outcomes in all sub-populations, age groups and geographic settings. To meet these targets, it is important that all vulnerable sub-populations including people with disabilities are not left behind in the HIV/AIDS response.

Basing on the International Classification of Functioning, Disability and Health (ICF) model (Cieza et al. 2009; Schwarzkopf, Grill & Dreinhöfer 2010; Üstün et al. 2003; WHO 2001), disability is not only having physical impairments. Functioning is defined in ICF model as an umbrella term for body functions, body structures, activities and participation. In this framework, a health condition may lead to an abnormality in body structure or function (i.e., impairment e.g., mobility), which can consequently cause activity limitations (e.g., difficulty walking) and participation restriction (e.g., exclusion from employment). This pathway will not be the same for all people as it is influenced by a range of personal factors (e.g., education) and environmental factors (e.g., terrain). Looking at disability using the ICF model, there are several ways it may affect knowledge on HIV prevention methods in an individual. Within the Ugandan settings, people with disabilities have not been adequately considered in HIV programmes (Schenk et al. 2020). As a result, people with disabilities may not be able to access information on HIV prevention methods compared to those without disabilities (Abimanyi-Ochom et al. 2017). Even if they were able to access this information, this would not be helpful to those with impairments in the domains of hearing, vision or cognition because the information used in most of the Ugandan settings is normally designed for people without disabilities. Because most of the information on HIV prevention methods is given during health education sessions at health care facilities, those with mobility problems and those who cannot access the health care facilities because of terrain may miss this information. People with disabilities within most of the African settings face economic hardships (Banks et al. 2017), and this may lead to a lack of transport to health care facilities where information on HIV prevention methods is usually given.

Although some work has been done on disability and HIV prevention methods in South Africa (Eide et al. 2011; Hanass-Hancock, Strode & Grant 2011; Rohleder 2010), currently, there is a paucity of data on disability and knowledge on HIV prevention methods in Uganda. This article adds on the scanty literature available on severity of disability and knowledge on HIV prevention methods in Uganda. We analysed available data from a single cross-sectional survey round of the General Population Cohort (GPC) study conducted in rural southwest Uganda to investigate the association between severity of disability and not having knowledge of any HIV prevention methods among adults.

## Research methods and design

### Study setting

The GPC study site is located in Kyamulibwa subcounty in Kalungu district, rural southwest Uganda. The GPC covers 25 neighbouring villages with approximately 23 000 residents. The main economic activity in the study setting is crop farming with a few households rearing animals. The predominant tribe is Baganda and the predominant religion is Catholicism.

### Study design

The current investigation analysed data from a single cross-sectional survey round of adult residents from 13 villages out of the 25 covered by the GPC study. While the entire GPC survey round (round 24) was conducted between January 2014 and November 2015, data on Washington group short set (WG-SS) on functioning (Groce & Mont 2017) was only collected between January 2015 until November 2015. The GPC study has been previously described (Asiki et al. 2013). In brief, the GPC is an open population-based longitudinal study that was established in 1989 to study the epidemiology of HIV in a rural African population following a collaboration between the Medical Research Council (MRC) UK and the Uganda Virus Research Institute (UVRI) (Asiki et al. 2013). However, over time, other research topics including disability, social science questions, NCDs and other emerging infections were included. The cohort runs a GPC clinic that offers free outpatient healthcare services for cohort participants. In addition, the cohort runs an HIV clinic that provides antiretroviral drugs for people living with HIV.

### Cohort measures

The study population is assessed through annual house-to-house census rounds and a biennial serological or medical survey, during which demographic, socio-medical and serological data are collected. Information gathered regularly includes data on fertility, mortality, migration, sexual behaviour, perceptions of HIV infection and HIV status. Other health-related information is also investigated, with topics varying annually.

Between January 2015 and November 2015 (the second half of round 24 of the GPC), we included questions on WG-SS on functioning (Groce & Mont 2017) to the survey questionnaire. In brief, the Washington group questions are a set of questions to identify people with disability. It contains multiple response questions on six domains of disability including difficulty performing basic universal activities such as walking, seeing, hearing, cognition, self-care and communication. The response values in each domain included: no difficulty, some difficulty, a lot of difficulty and cannot do at all.

### HIV status

Human immunodeficiency virus testing during round 24 was done following the Ministry of Health of Uganda testing algorithm. The algorithm for HIV rapid testing consisted of an initial screening with the rapid test, Determine HIV-1/2. If the test result was negative, the participant was given a negative diagnosis with no further rapid testing. If the test result was positive, the sample was retested with the rapid test HIV-1/2 Stat-Pak. If both tests gave a positive result, the participant was given a diagnosis of HIV-positive with no further rapid testing. If the tests gave discordant results (i.e., one positive and the other negative), the sample was further evaluated with the rapid test UniGold Recombinant HIV-1/2. For those samples assessed by all three tests, two positive test results were interpreted as a positive diagnosis. If two of the three tests gave negative results, then the participant was HIV-negative. The tests were performed by trained nurses who are part of the GPC study team. All the HIV rapid tests were performed from the field during the survey. However, positive tests and discordant tests were confirmed within the clinical laboratory by trained laboratory technicians. Within the GPC study team, there was a counsellor who did pre- and post-test counselling. For all positive tests, the results were communicated to the study participants by the counsellor following the Ugandan counselling guidelines.

### Statistical methods

#### Washington group short set on functioning

Disability was the main exposure of interest, which was defined based on a quantitative continuum of functioning classified into: ‘without disability’, ‘mild’, ‘moderate’ and ‘severe’ disability. The continuum was created by combining the multiple responses from the WG-SS questions across all the domains for each participant.

The ‘without disability’ classification comprised of participants who reported ‘no difficulty’ over all 6 domains. The ‘mild’ classification included participants with 1–4 domains reported as ‘some difficulty’ and no other domain coded as ‘a lot of difficulty or cannot do at all’.

The ‘moderate’ classification included participants with 5 or 6 functioning domains coded ‘some difficulty’ or up to 3 domains coded ‘a lot of difficulty’, but with no other domain coded ‘cannot do at all’. The ‘severe’ classification had participants with 4 or more functioning domains coded ‘a lot of difficulty’ or any other domain reported as ‘cannot do at all’.

#### Statistical modelling

Having no knowledge of any HIV prevention method was the outcome of interest, defined as a dichotomous variable. Logistic regression models were fitted for both the bivariate and multivariable models assessing the association between disability and not having knowledge of any HIV prevention method. Age and sex were considered *a priori* confounders. However, other factors were considered potential confounders and included in the multivariable model if there was a 15% change in the coefficients of disability after including that variable in the model.

Variables were included in the model based on a hierarchical relationship starting with the distal to the proximate factors. In turn, variables were added into the models as follows: age and sex, religion and marital status, education and current occupation, number of sexual partners in the last 12 months and other sexual behavioural factors, and HIV status in the fully adjusted model. Where variables were collinear, we selected the variable that explained confounding with disability. The fully adjusted model was assessed for collinearity based on variance inflation, and for effect modification by inclusion of interaction terms. The joint effects of having severe or moderate disability and none or incomplete primary education were assessed in comparison to their expected sum of individual effects given the likely contribution of education on knowing HIV prevention methods. Statistical significance was determined at 5% level.

### Ethical considerations

Ethical approval to conduct this study was obtained from the Uganda Virus Research Institute Research and Ethics Committee (No. GC/127/15/12/540) and from the Uganda National Council for Science and Technology (No. HS 640). We also obtained written or thumb-printed informed consents from each of the participants in this study.

## Results

### Socio-demographic and behavioural characteristics

A total of 3331 participants (60.4% were female) were asked on the WG-SS questions. Participants were aged at least 16 years for females and 18 years for males with a median age of 37 years in females (interquartile range [IQR]: 25–51) and 39 years (IQR: 24–52) in males. Among all participants, 18.9% (*n* = 628) were aged 55 years and above. At the time of the survey, over half of the participants were married (54.5%, *n* = 1815), a quarter (24.7%, *n* = 823) were never married/cohabited, while 9.0% (*n* = 299) were separated or divorced (Table 1). Among the participants, 38.0% (*n* = 1297) reported to have had no or incomplete primary education. Only 15.4% (*n* = 514) reported their main source of livelihood as something else other than agriculture, while 16.5% (*n* = 550) were unemployed.

**TABLE 1:** Participant characteristics and differences in severity of disability by each characteristic.

Characteristic	All participants		Severity of disability†					
	*n*	Column %	None		Mild		Moderate or Severe	
			*n*	Row %	*n*	Row %	*n*	Row %
Number of respondents	3331	-	1733	52.0	1116	33.5	482	14.5
**Sex**								
Male	1319	39.6	700	53.1	432	32.8	187	14.2
Female	2012	60.4	1033	51.3	684	34.0	295	14.7
**Age (in years)**								
16–25	894	26.8	684	76.5	165	18.5	45	5.0
26–35	614	18.4	442	72.0	130	21.2	42	6.8
36–55	1195	35.9	546	45.7	529	44.3	120	10.0
> 55	628	18.9	61	9.7	292	46.5	275	43.8
**Religion**								
Catholic	1867	56.0	947	50.7	640	34.3	280	15.0
Anglican	372	11.2	188	50.5	118	31.7	66	17.7
Other Christian	165	5.0	86	52.1	58	35.2	21	12.7
Muslim	927	27.8	512	55.2	300	32.4	115	12.4
**Marital status**								
Never married or cohabited	823	24.7	596	72.4	174	21.1	53	6.4
Currently married	1815	54.5	939	51.7	663	36.5	213	11.7
Widowed	394	11.8	154	39.1	156	39.6	84	21.3
Separated or divorced	299	9.0	44	14.7	123	41.1	132	44.1
**Education level completed**								
None or incomplete primary	1297	38.9	512	39.5	496	38.2	289	22.3
Completed primary and above	2034	61.1	1221	60.0	620	30.5	193	9.5
**Occupation**								
Cash crop cultivator	773	23.2	324	41.9	315	40.8	134	17.3
Subsistence crop	1494	44.9	713	47.7	537	35.9	244	16.3
Non-crop	514	15.4	331	64.4	145	28.2	38	7.4
Unemployed	550	16.5	365	66.4	119	21.6	66	12.0
**Age at first sex (in years)**								
Never	260	7.8	193	74.6	54	20.5	13	4.9
≤ 16	1379	41.4	660	47.9	498	36.1	221	16.0
17–19	1257	37.7	669	53.2	414	32.9	174	13.8
≥ 20	435	13.1	211	48.5	150	34.5	74	17.0
**Any current main partner**								
Yes	2094	62.9	1148	54.8	723	34.5	223	10.6
No	977	29.3	390	39.9	340	34.8	247	25.3
Never	260	7.8	195	75.0	53	20.4	12	4.6
**Last time of sex**								
< 1 month	1679	50.4	973	58.0	576	34.3	130	7.7
1–12 months ago	564	16.9	346	61.3	142	25.2	76	13.5
> 1 year ago	828	24.9	219	26.4	345	41.7	264	31.9
Never	260	7.8	195	75.0	53	20.4	12	4.6
**Number of partners in the last year**								
None	1059	31.8	403	38.1	390	36.8	266	25.1
1 partner	1887	56.6	1126	59.7	587	31.1	174	9.2
2 or more	385	11.6	204	53.0	139	36.1	42	10.9
**HIV test result**								
Negative	2997	90.0	1554	51.9	1009	33.7	434	14.5
Positive	334	10.0	179	53.6	107	32.0	48	14.4

*Source:* Groce, N.E. & Mont, D., 2017, ‘Counting disability: Emerging consensus on the Washington Group questionnaire’, *The Lancet Global Health* 5(7), e649–e650. https://doi.org/10.1016/S2214-109X(17)30207-3

† , Based on the Washington group short set (WG-SS) on functioning questionnaire.

Among all participants, 62.9% (*n* = 2094) reported having a current main partner, 7.8% (*n* = 260) had never had sex, while 50.4% (*n* = 1679) reported having had sex within the last 1 month. In addition, 11.6% (*n* = 385) reported at least two sexual partners in the last 12 months prior to the interview, while 56.6% (*n* = 1887) reported one sexual partner. This setting has a high HIV prevalence, with an overall HIV prevalence of 10.0% (*n* = 334) among participants, and women had a substantially higher prevalence than men (11.2% vs. 8.3%).

### Proportion with severe or moderate disability

Almost half of the population had some sort of disability (48.0%, *n* = 1598) categorised as either mild, moderate or severe. Disability was classified as severe or moderate in 14.5% (*n* = 482) of the population (13.3%, *n* = 443 with moderate disability and 1.2%, *n* = 39 with severe disability) and as mild in 33.5% (*n* = 1116). The largest portion in the overall severe or moderate disability was attributable to disability in cognition, movement and vision, where overall severe or moderate disability reduced by 4.3%, 3.9% and 3.6% when these domains were not considered in turn. Communication, self-care and hearing had lesser impact on the overall severe or moderate disability.

Disability increased with age (*p* < 0.001) (Table 1), and more exponentially among participants aged 55 years and above for both males and females. Among older participants aged 36 years and above, the proportion of disability was substantially higher in women than men (36–54 years: 11.5% vs. 7.8%; 55+ years: 46.7% versus 40.0% in women and men, respectively). However, among those aged 25 years and less, the proportion of moderate or severe disability was higher among young men than young women (7.1% in men vs. 3.6% in women).

After adjusting for age, severe or moderate disability was associated with being separated or divorced (*p* < 0.001), having none/incomplete primary education (*p* < 0.001), not currently having a main sexual partner (*p* < 0.001), more than a year without sex (*p* < 0.001), more than two sexual partners, no condom use at last sex with a casual partner (*p* = 0.001), and those who suffered a fall in the last year (*p* < 0.001). Those with occupations which did not involve crop growing were less likely to have severe or moderate disability (*p* = 0.002).

### Proportion without knowledge of HIV prevention methods

Overall, 4.2% (*n* = 140) of the respondents reported not knowing any HIV prevention method, with a slightly higher proportion among females than males (3.1% in males, 4.9% in females; crude odds ratio [OR] = 1.61; 95% Confidence Interval [CI]: 1.11–2.34; *p*-value = 0.011) despite no substantial differences observed in disability by sex. Severity of disability remained associated with increased likelihood of not knowing any HIV prevention method in the crude and adjusted models (*p* < 0.001). In the fully adjusted model, participants with moderate or severe disability were 5.5 times more likely not to know any HIV prevention methods (adjusted OR = 5.45, 95% Cl = 3.25–9.13) than those without any disability (Table 2). Even those classified with mild disability were associated with 62% higher odds of not knowing any HIV prevention method (crude OR = 1.62, 95% Cl = 0.99–2.64), although with borderline statistical significance (*p* = 0.054). The larger decrease in the effects of disability after adding the variables of education and occupation suggests that this set of variables have a largest impact on the relationship between disability and not knowing any HIV prevention method.

**TABLE 2:** Association between severity of disability and not having knowledge of any HIV prevention method among adult residents in the Kyamulibwa general population cohort.

Characteristic	Total		Without knowledge of any HIV prevention method		Adjusted odds ratio (95% confidence interval)				
	*n*	*%*	*n*	*%*	Age and sex	+Religion, marital status	+Education, current occupation	+Number of sexual partners in the < 12 months	+HIV status (Fully adjusted model)
*n*	%
**Overall**	3331	-	140	4.2	-	-	-	-	-
**Severity of disability†**									
None	1733	52.0	37	2.1	Reference	Reference	Reference	Reference	Reference
Mild	1116	33.5	42	3.8	1.87 (1.16–3.01)	1.86 (1.15–3.00)	1.71 (1.06–2.78)	1.64 (1.01–2.67)	1.62 (0.99–2.64)
Moderate or Severe	482	14.5	61	12.7	6.96 (4.24–11.45)	6.58 (3.98–10.87)	5.77 (3.46–9.62)	5.46 (3.27–9.14)	5.45 (3.25–9.13)
Likelihood ratio test *p*-value	-	-	-	-	*p* < 0.001	*p* < 0.001	*p* < 0.001	*p* < 0.001	*p* < 0.001

*Source:* Groce, N.E. & Mont, D., 2017, ‘Counting disability: Emerging consensus on the Washington Group questionnaire’, *The Lancet Global Health* 5(7), e649–e650. https://doi.org/10.1016/S2214-109X(17)30207-3

HIV, human immunodeficiency virus.

† , Based on the Washington group short set (WG-SS) on functioning questionnaire.

Our findings further suggest that the combined effect of having both severe or moderate disability and none or incomplete primary education was synergistic, given that their joint effect was 3.3 times higher (*p* < 0.001) than the sum of the individual effects of each (Online Appendix 1: Table 1-A1). In the fully adjusted model, sex and age did not remain independently associated with not knowing any HIV prevention methods (Online Appendix 1: Table 2-A1). On the other hand, occupation and education remained independently associated with not knowing any HIV prevention methods. The results did not show any suggestion for collinearity in the fully adjusted model and did not find interaction between disability and other independent factors, except education which was a strong confounder.

## Discussion

Findings from this study show that people with disabilities determined by the Washington group questions were almost five times likely not to know any HIV prevention method compared to those without disabilities. In addition, a higher proportion of women with disabilities reported not knowing any HIV prevention method compared to men. As the Joint United Nations Programme on HIV/AIDS aims for HIV testing, treatment and viral suppression rates to be 95%-95%-95% by 2025 (Heath, Levi & Hill 2021, McCreesh et al. 2017), it is important that no population group is left behind in this endeavour (Assefa et al. 2020), including people with disabilities.

For people living with HIV to join the cascade of care, they need to first have knowledge on HIV prevention methods, whose first step is to know one’s HIV status by undergoing HIV testing. The other methods for HIV prevention in Uganda include, having all people who test HIV-positive starting antiretroviral drugs immediately (Eisinger et al. 2019), voluntary male surgical circumcision (Loevinsohn et al. 2021; Reed et al. 2012), prevention of mother-to-child transmission (Schouten et al. 2011), pre-exposure prophylaxis (Burns et al. 2014) and post-exposure prophylaxis (Sultan, Benn & Waters 2014), and consistent condom use (Ahmed et al. 2001; Smith et al. 2015; Ukwuani, Tsui & Suchindran 2003). Combination HIV prevention strategies have also been recommended (Baxter & Abdool Karim 2016; Grabowski et al. 2017; Kremer et al. 2023). All population groups should have knowledge on these HIV prevention strategies, as a first step, towards HIV prevention and contribute to the global goal of ending the HIV epidemic as a public threat by 2030.

There are several things that may explain a lack of knowledge about HIV prevention among people with disabilities. These may include stigma and discrimination (Andersen 2006; Elliott, Utyasheva & Zack 2009; Jackson-Best & Edwards 2018; Schenk et al. 2020), health facility-related factors including inaccessible facilities (Nampewo 2017), a lack of access to affordable health and social services (Hanass-Hancock & Alli 2015) and social and economic factors (Banks et al. 2017; Braathen et al. 2016). For people with disabilities not to be left behind in the HIV fight, all these factors need to be looked at in both HIV programming and practice.

From our literature search, most of the longitudinal cohorts on HIV epidemiology have not adequately included questions on disability and HIV (Rohleder et al. 2009). Most of the studies which have been undertaken in SSA have been cross-sectional studies, and a few are demographic and health surveys. One of the strengths of this article is that for the first time within the cohort, we collected survey data on disability using the Washington group questions. Within the GPC, these data are important baseline data on HIV and disability, and will enable us to look at different time points in our cohort to see how knowledge on HIV in general and HIV prevention methods are evolving among people with disabilities.

This article also has some limitations. Firstly, we did not relate each individual disability with HIV prevention knowledge. To do this, a large enough number of participants with an individual disability would be needed. Some individual disabilities (e.g., deafness) have been shown to be highly associated with a lack of knowledge on HIV prevention methods (Groce, Yousafzai & Van Der Maas 2007; Groce et al. 2006). However, using the Washington group question is the best because there may be individuals with decreased functioning which may not be identified by looking at an individual disability. Secondly, we would like to acknowledge that data for this study were collected in 2015, and many things may have changed since then. However, this study brings in some evidence of a strong association between disability determined by the Washington group questions and HIV prevention knowledge. Within the GPC round we are conducting currently (running between 2023 and 2025 inclusive), we are again collecting data on Washington group questions which should enable us to look at differences on disability and HIV prevention knowledge in a period of one decade.

The findings from this paper call for effective HIV prevention strategies that consider the unique needs of people with disabilities and address their barriers to accessing HIV services.
